# Adaptive crossover designs for assessment of symptomatic treatments targeting behaviour in neurodegenerative disease: a phase 2 clinical trial of intranasal oxytocin for frontotemporal dementia (FOXY)

**DOI:** 10.1186/s13195-018-0427-2

**Published:** 2018-09-27

**Authors:** Elizabeth Finger, Scott Berry, Jeffrey Cummings, Kristy Coleman, Robin Hsiung, Howard H. Feldman, Adam Boxer

**Affiliations:** 1 Department of Clinical Neurological Sciences, Schulich School of Medicine and Dentistry, University of Western Ontario, London, ON Canada; 2 Parkwood Institute and Lawson Health Research Institute, 550 Wellington Road South, London, ON N6C 0A7 Canada; 3 Berry Consultants, Austin, TX USA; 4 Cleveland Clinic Lou Ruvo Center for Brain Health, Las Vegas, NV USA; 5 Department of Medicine, Division of Neurology, University of British Columbia, Vancouver, BC Canada; 6 Department of Neurosciences, Alzheimer’s Disease Cooperative Study, University of California, San Diego, CA USA; 7 Department of Neurology, University of California San Francisco School of Medicine, San Francisco, CA USA

**Keywords:** Oxytocin, Frontotemporal dementia, Empathy, Apathy, Adaptive design, Crossover design, Clinical trial

## Abstract

**Background:**

There are currently no treatments for empathy deficits in neuropsychiatric disorders. Acute administration of the hormone oxytocin has been associated with symptomatic improvements across animal models and several neuropsychiatric disorders, but results of the majority of oxytocin randomised controlled trials (RCTs) of longer duration have been negative or inconclusive. This lack of efficacy of may be due to rapid habituation to oxytocin with chronic dosing. The objective of the present study is to describe the design of a phase 2 adaptive randomised controlled crossover trial of intranasal oxytocin in frontotemporal dementia (FOXY) as an efficient model for future investigations of symptomatic treatments in neuropsychiatric and neurodegenerative disorders.

**Methods:**

Stage 1 will identify which of three dose schedules is most promising based on change in the primary outcome measure, the Neuropsychiatric Inventory apathy/indifference domain score, over 6 weeks of treatment. In stage 2, additional patients are enrolled at the most promising dose for preliminary efficacy analysis when combined with stage 1 to determine if a phase 3 trial is warranted. Objective measures include facial expression recognition, cerebrospinal fluid (CSF) oxytocin levels, and behavioural ratings of videotaped interactions.

**Results:**

A total of 20 patients per arm will be entered into stage 1 for a total of 60 patients. In stage 2, an additional 40 patients will be enrolled in the most promising dose arm.

**Conclusions:**

The use of adaptive, crossover designs and inclusion of objective measures of change in CSF oxytocin levels and social behaviour will improve the efficiency and conclusiveness of RCTs of oxytocin and other symptomatic treatments in neuropsychiatric disorders.

**Trial registration:**

ClinicalTrials.gov, NCT03260920. Registered on August 24, 2017.

**Electronic supplementary material:**

The online version of this article (10.1186/s13195-018-0427-2) contains supplementary material, which is available to authorized users.

## Background

Social apathy and loss of empathy are hallmark features of frontotemporal dementia (FTD), for which there are currently no approved or effective treatments. Oxytocin, a neuropeptide modulating social behaviour across species, has been identified as a potential symptomatic therapy for empathy and related social behaviour impairments across neuropsychiatric conditions [1]. However, to date, the evidence supporting the long-term use of oxytocin is lacking. Identification of effective, evidence-based symptomatic treatments for social cognition and behaviour deficits in FTD and other neuropsychiatric disorders raises several unique challenges for clinical trial design and implementation. These challenges include symptom and behavioural heterogeneity, the nonlinear trajectory of many behavioural symptoms over the course of the disease, lack of harmonisation of assessment and outcome measures across centres, and reliance on subjective caregiver reports for key outcome measures [2–4]. Additional challenges for randomised controlled trials (RCTs) of oxytocin include potential differential responses according to sex, uncertainties around brain penetration of intranasal formulations, lack of dose-finding studies, and confirmation of target engagement [5].

Further, specific to oxytocin, although numerous published reports cite improvements in social cognition in several disorders following single-dose administration [6–8], longer-duration (2–6 weeks) RCTs of oxytocin have had mixed results, with several showing no or small effects [9–13]. These trials have used once- or twice-daily dose schedules and have not included design elements to address the potential habituation of responses with chronic dosing that has been reported in non-human animal studies [14–17]. In patients with FTD, short-term administration of oxytocin was associated with improvement in caregiver ratings of social behaviours and effects of emotional facial expression recognition [18, 19], though longer-term studies have not yet been conducted. A formal dose-finding study in FTD identified 72 IU twice daily as the most feasible dose, a dose larger than that used in the majority of oxytocin RCTs in other disorders [19].

To address the lack of specific symptomatic treatment in FTD and limitations of oxytocin and related trial designs targeting social behaviours across a range of disorders to date, we describe a novel adaptive proof-of-concept, phase 2, placebo-controlled, randomised crossover trial repurposing the hormone and neuropeptide oxytocin as a potential symptomatic treatment for apathy/indifference and related empathy deficits in patients with FTD. The objectives of the study design are to (1) efficiently identify the most promising dose schedule of oxytocin, given potential habituation to daily dosing, and (2) permit efficacy analysis of the most promising dose compared with placebo to determine whether progression to a phase 3 trial is warranted. We propose that this approach may inform the design and conduct of other RCTs, particularly of symptomatic medications in FTD and related neuropsychiatric disorders.

## Methods

### Participants

Participants with a diagnosis of probable FTD (behavioural variant FTD, FTD semantic subtype or FTD progressive non-fluent aphasia) [20, 21] with current symptoms of social apathy/indifference as measured by Neuropsychiatric Inventory (NPI) apathy/indifference severity subscale score ≥ 2 [22], supportive brain imaging based on centrally rated frontotemporal atrophy score of ≥ 2 based on brain magnetic resonance imaging or computed tomography [23] or FTD pattern of hypometabolism on fluorodeoxyglucose positron emission tomography or hypoperfusion on single-photon emission computed tomography, or known causal genetic mutation, and a caregiver who sees the patient daily for at least 3 h/d and who can administer all trial medications are eligible for the study. Additional inclusion criteria include a frontotemporal lobar degeneration Clinical Dementia Rating (FTLD-CDR) [2] score consistent with mild or moderate dementia, Mini Mental State Examination score > 10/30, and stable baseline medications for ≥ 30 days. Exclusion criteria include recent myocardial infarction, congestive heart failure, current uncontrolled hypertension, bradycardia, long QTc or hyponatremia. Participants will be enrolled at one of ten FTD centres in the United States and Canada comprising the Advancing Research & Treatment for Frontotemporal Lobar Degeneration network (www.rarediseasesnetwork.org/cms/artfl) plus three additional Canadian sites.

### Study design

**Fig. 1:**
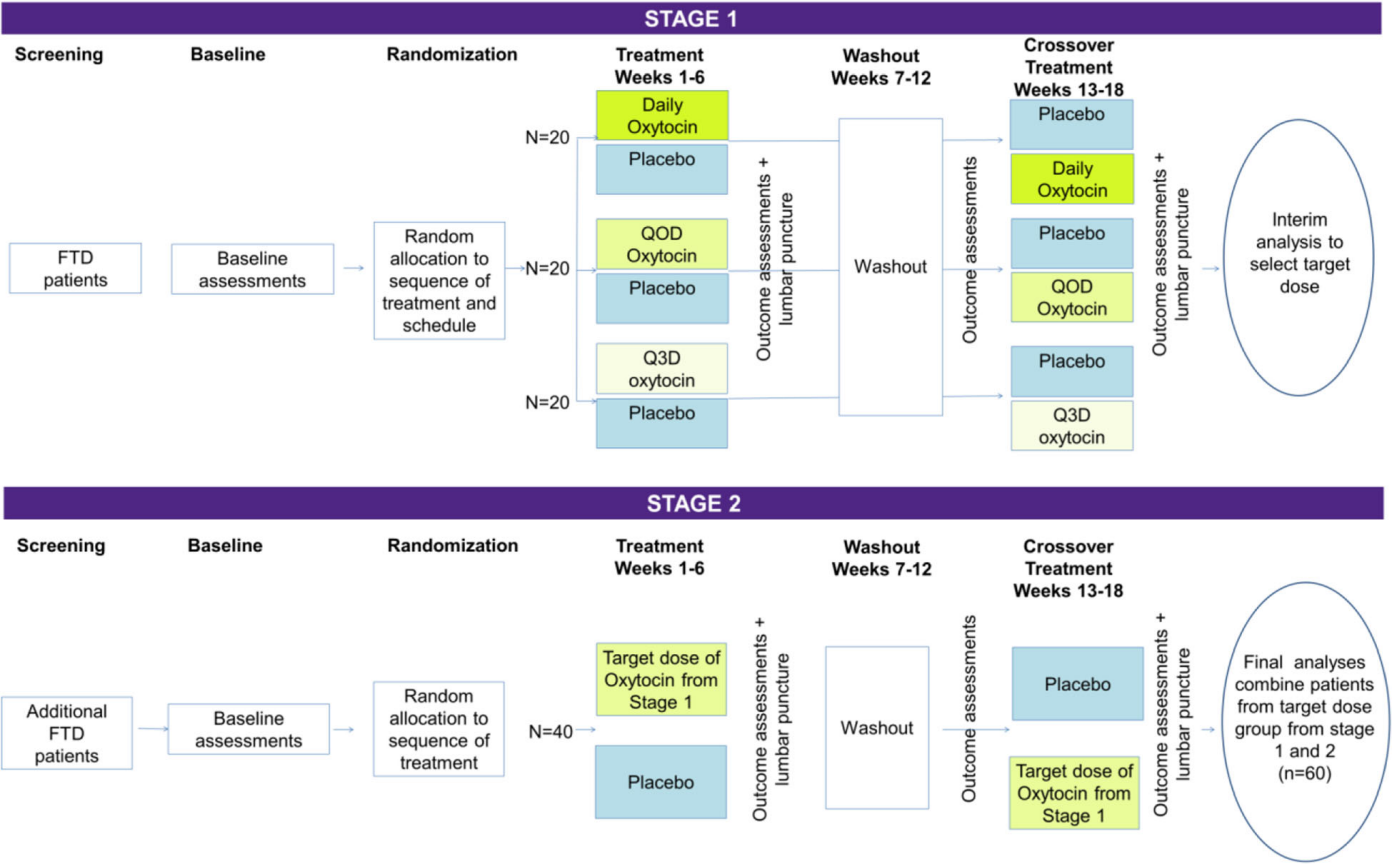
Two-stage phase II adaptive crossover trial design for intranasal oxytocin for frontotemporal dementia (FOXY). In stage 1, a total of 60 patients with frontotemporal dementia (FTD) are randomized to one of three dose schedules. In the crossover design, baseline assessments are completed at the beginning of each treatment period. After baseline, participants receive twice-daily intranasal sprays of placebo or oxytocin for 6 weeks and then undergo complete outcome assessments and optional lumbar puncture. The first treatment period is followed by a washout period with no sprays given for 6 weeks. At the end of the washout period, participants are re-baselined prior to 6 weeks of twice-daily intranasal spays of the alternate drug (placebo or oxytocin). In stage 2, 20 additional patients with FTD are randomized to the most promising dose identified at the planned interim analyses at the end of stage 1, and complete procedures identical to those in stage 1 are performed

The study design features a placebo-controlled, randomised crossover trial comparing changes in social apathy and empathy following 6 weeks of oxytocin treatment with 6 weeks of placebo, with a 6-week washout between periods (Fig. 1). Although FTD is a progressive disorder, changes in apathy over this interval are predicted to be small; over an 8-week longitudinal study of patients with FTD, the mean change in apathy ratings on the Frontal Behavioral Inventory [24] was 0.13% (range − 6% to +7%) [25]. In stage 1 the trial will compare three dosing schedules of 72 IU intranasal oxytocin (daily, alternate days, or every third day dosing) for patients with FTD compared with placebo. At the end of stage 1, a Bayesian analysis will be conducted to identify the most promising dose schedule, termed the ‘target’ dose schedule. In stage 2, forty additional patients will be enrolled at the target dose schedule. In both stages the primary outcome measure is mean change from baseline on the Neuropsychiatric Inventory (NPI) apathy/indifference domain score comparing on-active versus on-placebo. At the end of stage 2, data from patients receiving the target dose (from stage 1 and stage 2) are combined in the efficacy analysis. An optional substudy measuring cerebrospinal fluid (CSF) oxytocin levels at the end of the oxytocin and placebo treatment periods will confirm CSF oxytocin level rises in FTD and determine whether changes in CSF oxytocin levels correlate with behavioural measures. The three dosing schedules in the proposed study were selected on the basis of (1) a prior dose-finding study of intranasal oxytocin in FTD identifying 72 IU as the most promising feasible dose [19], (2) estimated half-life of oxytocin in the central nervous system (CNS) [26–28], and (3) predictions that dosing < 2 d/wk would be unlikely to result in a clinically meaningful effect.

### Randomisation

Randomisation for stages 1 and 2 will be stratified across the treatment groups listed above according to sex and disease severity (mild vs. moderate) because oxytocin is known to have differential behavioural effects based on sex [29], and pilot data suggest that efficacy may differ as a function of disease severity and the integrity of remaining oxytocin receptor-positive neurons [19, 30]. The FTLD-CDR allows severity assessment and stratification across the different FTD phenotypes included in the trial. The number of centres required for the trial precludes stratification according to centre. Participants will be randomised using variable block sizes concealed from participating sites.

### Outcome measures and minimum clinically significant difference

The NPI apathy/indifference domain score is the designated primary outcome, with a ≥ 2-point improvement on the NPI identified to be clinically significant and to represent meaningful improvement in patient symptoms of apathy and loss of empathy. A 2-point minimal clinically important difference is consistent with prior trials using the NPI in FTD [31, 32]. In the pilot study of oxytocin in FTD, a 2-point improvement corresponded to a reduction of approximately 30% in apathy/indifference ratings. The NPI apathy domain score was selected as the primary outcome measure because it was where we saw the most significant differences in our pilot study [19]. In the pilot study no significant differences were observed on the Apathy Evaluation Scale, which we attributed to the lack of items indexing increased conversations or empathic behaviours towards family members, and several items related to insight, which is impaired in FTD and not expected to be remedied by oxytocin. Compared with other available measures, the NPI prompts for the caregiver are holistic enough to capture both social and non-social apathy and interactions with others that we hypothesised may be modulated by oxytocin, as well as to capture both the severity and frequency of such behaviours within the domain score. Secondary outcome measures included change in the Interpersonal Reactivity Index empathic concern score [33], NPI caregiver distress scores [22], and the Revised Self-Monitoring Scale [34]. Accuracy of emotional facial expression recognition and blinded ratings of naturalistic videotaped behaviours of patients as they have a meal with their caregivers using the Social Observation Checklist [35] will serve as a measure of pharmacodynamic effects (Table 1). Difference in CSF levels of oxytocin following the oxytocin vs. placebo treatment period will be examined to confirm entry of intranasally administered oxytocin into the CSF. Potential adverse symptoms will be monitored, and changes in serum sodium level, heart rate, QTc and blood pressure will be assessed at baseline and the beginning and end of each treatment period. Compliance with treatment will be monitored with daily caregiver-completed administration logs and measurement of residual volumes.

**Table 1:** Outcome assessments completed at baseline assessment, end of treatment period 1, after washout (baseline 2), and end of treatment period 2

Primary outcome measure:	
• Neuropsychiatry Inventory (NPI) apathy/indifference domain score [42]	
Secondary outcome measures:	
• Interpersonal Reactivity Index (IRI) empathic concern scale and total score [33]	
• Clinician’s Global Impression of Change (apathy) (m-CGIC) scores [43, 44]	
• Emotional facial expression recognition performance [17]	
• Revised Self-Monitoring Scale (RSMS) score [45]	
• NPI caregiver distress scores on NPI apathy/indifference scale and total caregiver distress scores [42]	
• Total NPI scores	
• Cambridge Behavioural Inventory [46]	
• Addenbrooke’s Cognitive Examination III [47]	
• Social Observation Checklist blinded central ratings of videotaped meals with patients and caregivers [35].	

### Data analysis and pre-specification of adaptive design decision algorithms

#### Stage 1

At the completion of stage 1 the analysis described below will take place to select the most promising dose schedule that will show the largest estimated mean change (benefit) of oxytocin relative to placebo on the primary outcome measure, the NPI apathy/indifference domain scale scores. A linear model with covariates for sex and order of treatment in the crossover will be used to estimate the efficacy of each treatment arm. Each patient will have a 6-week post-treatment change from baseline score for each treatment phase (i.e., week 6 − week 0 vs. week 18 − week 12). The within-patient difference in these values ‘on active’ vs. ‘on placebo’, Y, will be modelled assuming a normal linear model: *Y*~*N*(*θ*_*t*_ + *β*_1_*X*_1_ + *β*_2_*X*_2_, *σ*^2^), where *X*_*1*_ is an indicator of the patient having the active as the first treatment in the crossover and *X*_*2*_ an indicator of the patient being a male. The efficacy of the three active treatment arms are captured by the mean change in total NPI apathy/indifference treatment parameters, *θ*_*1*_, *θ*_*2*_, and *θ*_*3*_.

The treatment arm with the largest estimated mean change (benefit) will be selected for stage 2. Note that the dosing strategy selection analysis does not depend on a formal hypothesis test. If two or more dose schedules appear equally promising (i.e., similar estimated mean change), consideration of side effect profile can be used to select the most promising and tolerable dose schedule. If two or more dose schedules appear equally promising and their side effect profiles are similar, the more frequent dose schedule will be selected in stage 2. Enrolment in the groups and subgroups is relatively small, and important information may be gained by CSF oxytocin measurements as well as secondary outcome measures to inform future studies, thus no futility analysis will be conducted at the end of stage 1.

#### Stage 2

Given the crossover design and proof-of-concept nature of the study, a variant of a per-protocol analysis will be conducted on the patients completing the protocol, defined as those who entered into both phases of the treatment, have a primary outcome measure, and took ≥ 70% of sprays. A modified intention-to-treat analysis will be conducted as a supportive analysis, including all patients who were randomised and received at least one dose of study drug. Because the primary outcome measure can be obtained via telephone interview with the caregiver, wherever available, this will be collected for patients who do not complete the study, with the last observation carried forward.

## Results

### Sample size justification

#### Primary outcome measure

Sample size and clinical trial simulations were based on a published pilot study of oxytocin in FTD and designated minimal clinically significant difference of 2 points on the NPI apathy/indifference score [19]. Simulations were conducting using the Fixed and Adaptive Clinical Trial Simulator (www.berryconsultants.com) and varying the number of patients and effect sizes in stage 1 to show the operating characteristics. The simulations assumed an SD of 3.3 between the placebo and active arms (based on prior published studies of mean differences and SD on the NPI for an individual patient) [18]. Differences from 0 to 3 in the effect of an arm relative to placebo were explored for each trial design. For each design scenario 10,000 simulated trials were conducted. These simulations demonstrate that for a mean difference of 2 points or more from placebo, a sample size of 54 patients receiving the target dose schedule has an 86% probability of showing superiority to placebo at the end of stage 2 (Additional file 1). To enrol at least 54 patients in the target dose schedule, a total of 100 patients will be enrolled in this trial. In stage 1, 20 patients with FTD will be randomised to each of the three arms (daily, alternate days, or third-day dosing). In stage 2, an additional 40 patients will be randomised to the target dose schedule, resulting in a sample size of 60 patients at the target dose schedule across both study phases. With enrolment of 60 patients (30 males and 30 females) in the target dose schedule, subgroup analysis based on sex will have a power of 80% to detect a 2-point difference on the NPI apathy domain based on within-patient SD of differences of 3.3 in our pilot study [19] and will permit up to 15% loss of data due to potential non-compliance or loss to follow-up. For the main secondary outcome measure of interest, the empathic concern scale of the IRI, based on an SD of the change from baseline of 1.75 [19], a sample of 20 patients per arm per sex provides power of 80% to detect a 1-point difference.

#### CSF substudy

Following a study of a single dose of 24 IU intranasal oxytocin in which researchers found statistically significant increases in the adult volunteers’ CSF oxytocin measured at 75 min (+ 64%) [26], using paired *t* tests, a sample of 10 participants from the daily oxytocin dose schedule group provides power > 0.95 to detect a significant difference in CSF oxytocin levels after 6 weeks of oxytocin vs. placebo treatment.

## Discussion

The proposed trial represents an application of an adaptive crossover Bayesian design to improve the efficiency of determining the efficacy of symptomatic treatment for FTD. Specifically, and building on the Co-Enzyme Q10 in Amyotrophic Lateral Sclerosis study [36], the two-stage design permits dose schedule selection and efficacy assessment with a smaller sample size than traditional designs. The design addresses potential limitations of prior RCTs evaluating the effects of oxytocin on behaviour in other neuropsychiatric disorders by use of a crossover design, inclusion of an objective rating of videotaped naturalistic behaviours during each treatment phase, and measurements of CSF oxytocin levels to confirm entry of drug into the CNS. Inclusion of symptom-specific and global measures of caregiver distress will enable identification of meaningful clinical change for each measure for the present study as well as future interventional studies.

Bayesian adaptive designs are increasingly employed in state-of-the-art clinical trials [36, 37] and are endorsed by the U.S. Food and Drug Administration (FDA) and Patient-Centered Outcomes Research Institute. Adaptive designs are particularly helpful when there are multiple goals in the trial, such as finding the best dose schedule and confirming its efficacy. Extensive trial simulations have been used to compare different adaptations and parameters to select the most effective and efficient design. Advantages of this Bayesian adaptive design include smaller sample size to determine which treatment is the most effective, a reduced delay in identification of ineffective treatments, and decreasing the time to trial conclusions with the seamless shift between study phases/goals (i.e., dose schedule finding to efficacy). These are efficiencies commonly seen in adaptive trials [38]. Compared with traditional clinical trial designs, use of an adaptive design for the present study results in a 20% reduction in the number of patients needed (Table 2).

**Table 2:** Efficiencies in sample size of adaptive crossover trial compared with traditional and parallel trial designs

Study design	Sample size dose selection^a^	Sample size POC efficacy	Total sample size	Power
Traditional parallel arm^b^	62	44	106	0.86
Traditional crossover	54	44	106	0.86
Adaptive crossover	60	24	84	0.86

^a^Power calculations performed using G*Power were based on an effect size from a minimum clinically significant difference of 2 points on the NPI apathy/indifference domain score (*d* = 0.7) and power of 0.80 to permit analysis for males and females, with effect size of 0.7 based on pilot study results of a 3-point difference (*d*_karr_ = 0.7) [11]. For comparison, sample sizes assuming a smaller effect size are shown for each design

^b^Traditional trial design-based on factor design (analysis of covariance). Four groups (three dose schedules + placebo), *df* = 3, disease severity as covariate, with sample size doubled to permit separate analysis of males and females

Traditional crossover design comprised three groups, *df* = 2, disease severity as covariate, with sample size doubled to permit separate analysis of males and females

Recent RCTs of intranasal oxytocin for social cognition deficits in autistic spectrum disorders have had mixed results [3, 10]. Indications that caregiver assessments of change in behaviour are strongly correlated with assumptions about treatment status [3] have led the National Institute of Mental Health to specify that objective measures of treatment response should be included in studies of oxytocin in patient populations [39]. Given the lack of insight in patients with FTD and reliance on caregiver judgments regarding behavioural change, the same potential confound exists for behavioural outcome measures in FTD. Recent development and validation of the Social Observation Checklist using videotaped encounters between patients and caregivers during meals [35] provides an objective means by which to quantify naturalistic behaviours and reduce site-related variability by use of a blinded central rater. Use of a crossover design is also important in disorders such as FTD, where caregiver measurements are an important outcome measure and when significant subject-to-subject symptom heterogeneity is present. For example, all patients enrolled will meet inclusion criteria for social apathy and empathy deficits, the main behavioural symptoms potentially impacted by oxytocin, but patients are expected to have different combinations of other FTD behavioural symptoms (i.e., impulsivity, obsessive-compulsive behaviours, aggression) which could differentially interact with the effects of interest. The crossover allows the analysis to account for the subject-to-subject heterogeneity explicitly, greatly reducing the SD across subjects. Inclusion of oxytocin CSF measurements will provide needed data to determine whether CSF level changes correlate with behavioural changes, and thus inform dosing strategies. In the event that the clinical outcomes are positive, these data may help to refine dose selection for a phase 3 trial. In the event that there is no efficacy signal, the data would confirm that sufficient drug reached the CNS and therefore would support a negative (as opposed to inconclusive) result for oxytocin.

### Limitations

Currently, a direct measure of oxytocin target engagement in humans is limited by the lack of an available positron emission tomography tracer. Although the CSF measurements should aid in distinction between a negative trial and an inconclusive trial due to lack of drug in the CNS, it is possible that oxytocin levels may rise in specific brain regions proximal to the site of administration (basal forebrain, ventral frontal and mesial temporal lobes) or secondary to trigger of endogenous oxytocin release without significant rises in CSF [40]. However, if CSF oxytocin levels do not show measurement increases and the objective performance measures do not show efficacy, this would indicate that a phase 3 trial in FTD is not warranted. The present study will also address whether a 2-point improvement on the NPI, which has been used in prior clinical trials [31, 41], reflects a clinically significant reduction in caregiver distress. These critical indicators of the value of potential treatments for FTD will be assessed with the NPI caregiver distress scores. If results of this RCT are positive, knowledge of how a change in NPI scores corresponds to caregiver distress scores will critically inform designation of a minimal clinically significant difference for a phase 3 trial and FTD more globally.

## Conclusions

Adaptive design and crossover trials are uncommon designs in clinical trials in dementia. Building on the use of adaptive trial designs to assess potential neuroprotective treatments in neurodegenerative disorders [36], we describe an application of an adaptive crossover design to facilitate dose selection and efficacy assessment for symptomatic treatment in FTD. The design and inclusion of objective measures to index outcomes related to behaviour and emotion can be applied to study of oxytocin and other symptom-focused treatments in other neuropsychiatric and neurodegenerative disorders.

## Additional file

Additional file 1: Bayesian adaptive design trial simulations for FOXY. (DOCX 20 kb)
