# Supplementary material for: Adaptive crossover designs for assessment of symptomatic treatments targeting behaviour in neurodegenerative disease: a phase 2 clinical trial of intranasal oxytocin for frontotemporal dementia (FOXY)
Source: Alzheimers Res Ther. 2018 Sep 27;10:102. doi: 10.1186/s13195-018-0427-2 (PMC6161323; doi:10.1186/s13195-018-0427-2)
Supplement: Supplementary file 1 — Bayesian adaptive design trial simulations for FOXY. (DOCX 20 kb) [file 13195_2018_427_MOESM1_ESM.docx]

**Appendix 1: Bayesian Adaptive Design Trial Simulations for FOXY**

Simulations were conducting by Berry Consultants using the FACTS -- Fixed and Adaptive Clinical Trial Simulator and varying the number of patients and effect sizes in Stage 1 to show the operating characteristics. The simulations assumed a standard deviation of 3.3 between the placebo and active arm (based on our prior published studies of mean differences and SD on the NPI), for an individual patient. Differences from 0 to 3 in the effect of an arm are explored for each trial design. In each row (each design by scenario) 10,000 simulated trials were conducted. Among the various trial simulations we have selected the following design detailed below in which n=20 patients are randomized into each dose arm in stage 1 and an additional n=40 patients randomized into stage 2 to best balance power and efficiency. The following simulation results are assuming

- Total sample size of 100
- Stage 1: 20:20:20
- Stage 2: 40 to target arm
- Assumes one arm is the best, second has 50% of max arm, third has 25% of max arm
  - That best arm for ease is Arm 1 (but symmetric across arms)
- Final analysis compare target arm to placebo, including all Stage 1 and Stage 2 patients

| Arm | True Mean | Selected  Target | Win  Stage 2 | Mean N |
| --- | --- | --- | --- | --- |
| 1 | 0 | 0.334 | 0.014 | 33.4 |
| 2 | 0 | 0.332 | 0.017 | 33.3 |
| 3 | 0 | 0.334 | 0.015 | 33.4 |
| 1 | 0.5 | 0.457 | 0.13 | 38.3 |
| 2 | 0.25 | 0.305 | 0.046 | 32.2 |
| 3 | 0.125 | 0.238 | 0.022 | 29.5 |
| 1 | 1 | 0.579 | 0.411 | 43.2 |
| 2 | 0.5 | 0.264 | 0.092 | 30.5 |
| 3 | 0.25 | 0.158 | 0.03 | 26.3 |
| 1 | 1.5 | 0.694 | 0.663 | 47.8 |
| 2 | 0.75 | 0.211 | 0.13 | 28.4 |
| 3 | 0.375 | 0.095 | 0.03 | 23.8 |
| 1 | 2 | 0.788 | 0.786 | 51.5 |
| 2 | 1 | 0.159 | 0.132 | 26.4 |
| 3 | 0.5 | 0.053 | 0.026 | 22.1 |
| 1 | 2.5 | 0.86 | 0.86 | 54.4 |
| 2 | 1.25 | 0.114 | 0.11 | 24.6 |
| 3 | 0.625 | 0.026 | 0.018 | 21 |
| 1 | 3 | 0.911 | 0.911 | 56.4 |
| 2 | 1.5 | 0.077 | 0.077 | 23.1 |
| 3 | 0.75 | 0.012 | 0.01 | 20.5 |

For example, if we use the 2-stage design described above and one arm is at the clinically significant difference of 2 (circle/ true mean 2.5) and the other two arms have 50% (true mean 1.25) and 25% (true mean .625) benefit respective, then the good arm, arm 1, will be selected as the target dose 86% of the trials. That target dose will be found statistically superior to placebo 86% of the time it goes to stage 2 with a mean sample size of n=54. In 11% of the trials arm 2 and in 2.6% arm 3 will be selected – which is the wrong decision, but the randomness of the data in Stage 1 makes them look better. In these cases, rarely (11% or 1.8% respectively of trials) they will be shown superior.
